# Implementing Mindfulness in General Life and Organizations. Validation of the Time Flow Mindfulness Questionnaire for Effective Health Management

**DOI:** 10.3389/fpsyg.2022.832784

**Published:** 2022-04-07

**Authors:** Laura Petitta, Emanuela Sinato, Maria Teresa Giannelli, Miriam Palange

**Affiliations:** ^1^Department of Psychology, Sapienza University of Rome, Rome, Italy; ^2^Independent Researcher, Rome, Italy; ^3^Scuola di Specializzazione in Psicologia Clinica, Sapienza University of Rome, Rome, Italy

**Keywords:** new mindfulness questionnaire, time-centered, job burnout, work settings, multi-design validation

## Abstract

The primary purpose of the current research was to examine the psychometric properties of the Time Flow Mindfulness Questionnaire (TFMQ), a new self-report scale designed to measure cognitive, emotional, bodily, context-related, and action-related distracting inputs experienced by the mind during three different time windows of mindfulness practice (preliminary moments, during-the-practice, after-the-practice). The 42-item scale assesses the following second-order and first-order factors: Practice (preliminary, during), Benefits (short-term, long-term) and Benefits at work. Three studies were conducted. The first study assessed the factor structure and internal consistency on a sample of 141 mindfulness practitioners. Using a two-wave lagged design on a different sample of 46 trainees attending mindfulness based stress reduction (MBSR) courses, the second study examined concurrent validity by performing correlations between the TFMQ and Five Facets Mindfulness Questionnaire (FFMQ). The third study (same sample as Study 1) examined criterion validity by testing a structural equation model wherein mindfulness practice predicts job burnout, both directly and indirectly through mindfulness benefits at work. All studies relied on anonymous surveys. Our findings suggest that the TFMQ: (a) has a factor structure consistent with the hypothesized conceptual dimensions; (b) has good concurrent validity as demonstrated by significant correlations with the FFMQ dimensions; and (c) consists of mindfulness dimensions that predict job burnout in organizations (i.e., criterion validity). The TFMQ is a valid and reliable mindfulness measure that may help (a) practitioners gain awareness of different types of inputs that potentially distract the mind and mindfulness beneficial consequences, and (b) organizations implement mindfulness in work-settings.

## Introduction

The concept of mindfulness refers to the ability to attend to experiences (internal and external to the person) occurring in the present moment in both a non-evaluative and accepting way (Kabat-Zinn, 1990; Brown and Ryan, 2003). An overarching view of mindfulness can be summarized as a process of regulating one’s attention with the aim of approaching experiences with an open and non-judgmental awareness, recognizing and accepting the cognitive, emotional, bodily and environmental stimuli brought into consciousness and distracting one from being fully present in the moment (Kabat-Zinn, 1990; Walach et al., 2006). According to Gunaratana (1990, p. 74), distraction during the practice can be anything: “a sound, a sensation, an emotion, a fantasy, anything at all.” As such, training one’s mind to observe and accept distractions requires developing the ability to become aware of many different types of stimuli. Practicing mindfulness is one way to learn such ability to enter a state of mindfulness. Toward this end, mindfulness questionnaires are tools designed to measure the state of mindfulness experienced by a practitioner that may assist individuals who wish to track their progress by reflecting back on their experience and learning something new about themselves and their ability to meditate.

The current paper seeks to present a new mindfulness questionnaire, the Time Flow Mindfulness Questionnaire (TFMQ). The reason for proposing this new tool is threefold. First, a reasonably comprehensive measure of mindfulness should capture the individual’s ability to become aware of all sorts of distractions including cognitive, emotional, bodily, environmental, social and behavioral (e.g., impulse to react) stimuli. However, an overview of well-established self-report mindfulness questionnaires suggests that each tool generally aims at capturing only some components or dimensions of mindfulness (e.g., only selected facets of awareness and/or selected spokes of awareness; Siegel, 2018). Therefore, existing mindfulness scales mainly focus only on some of the above types of stimuli or a selected combination of them (Bergomi et al., 2013b; Ackerman, 2020). Consequently, to date, no mindfulness scale includes a measurement of all potential sources of distraction (i.e., cognitive, emotional, bodily, environmental, social and behavioral stimuli) let alone of all dimensions of mindfulness. Second, the literature (Gunaratana, 1990) suggests that mindfulness practice floats across temporally subsequent steps and flows across moments that range from preparatory moments throughout the practice and completion activities, up to and including moments that are both short-term and long-term subsequent to a mindfulness session and that allow the individual to gauge the level of mindfulness experienced and the potential well-being consequent to the practice (Siegel, 2007; Ackerman, 2020). Notwithstanding, to date, this time flow approach to mindfulness practice that captures the different sensations experienced by the individual across different temporal windows and activities of mindfulness practice, has never been operationalized. Third, given the widespread application of mindfulness in various clinical and health-related fields (Bergomi et al., 2013b; Ackerman, 2020), all renowned mindfulness scales were developed for general and/or clinical population. To date, no scale is designed to include different sections that aim at measuring the mindfulness experience and its beneficial consequences in both the general life of the individual as well as the work context.

Consistently, the current paper aims at studying the psychometric properties of the TFMQ, a new mindfulness questionnaire that seeks to address and overcome the literatures’ shortcomings related to the current self-report measures of mindfulness. Specifically, the TFMQ was developed in order to: (1) extend the comprehensiveness of the mindfulness aspects/dimensions and potentially distracting stimuli included in a mindfulness scale; (2) introduce a time flow approach to mindfulness measurement in order to capture the temporal stages of the individual’s experience of mindfulness practice (and its consequences), ranging from preparatory moments throughout the practice up to and including the investigation of how the practitioners feel in the moments following a mindfulness session; and (3) contextualize the mindfulness measurement to both the fields of individual’s daily and work-life. Toward this end, we utilized a multi-sample and multi-design approach in order to rely on independent samples and different research designs to test the multiple types of validity of the new questionnaire and draw our conclusions. Specifically, we addressed the following types of validity of the TFMQ throughout the three studies. Study 1 addressed the *internal validity* of the new questionnaire and thus, relied on the sole administering of this measure at one point in time (i.e., cross-sectional sampling). To this end, the dimensionality of the new questionnaire was assessed by performing Exploratory and Confirmatory Factor Analyses on cross-sectional data. Study 2 addressed *concurrent validity* in order to assess whether the new questionnaire actually measures mindfulness dimensions as proposed by a previous well-established measure of the construct and thus, included two different measurement scales. Toward this end, we used a longitudinal two-wave sample in order to test whether the correlations among the dimensions of the two different tools held at two different points in time, thus strengthening our conclusions. Finally, *criterion validity* was addressed in Study 3 in order to test whether the new mindfulness questionnaire contributes to predicting a health-related outcome (i.e., job burnout). To this end, we used the same cross-sectional sample of Study 1 in order to rely on a large sample that provided the necessary statistical power to perform Structural Equation Modeling (SEM) analyses on the hypothesized nomological network.

We begin with an overview of the theoretical foundations we draw upon in establishing our comprehensive and time flow approach to mindfulness measurement and developing the new TFMQ. Next, we briefly define job burnout and delineate arguments in order to formulate hypotheses regarding the impact of mindfulness on burnout and lay the foundation to test the criterion validity of the new scale. Finally, we present three validation studies of the TFMQ using a multi-sample and multi-design (i.e., cross-sectional, time-lagged). Specifically, studies one and two test respectfully the construct and concurrent validity on cross-sectional and time-lagged data whereas study three assesses the criterion validity by testing the hypothesized nomological network among mindfulness dimensions and job burnout on a sample of workers from different organizational settings.

### A Time-Centered Approach to Mindfulness and Its Relevance for Well-Being

According to the literature (Dorjee, 2010), the notion of mindfulness may be different depending on the components and mechanisms that are mainly involved in its study and examination. Consistently, the existing mindfulness scales differ with respect to fundamental aspects of the mindfulness construct they operationalize and, therefore, aspects that they capture (Bergomi et al., 2013b). Below we briefly review the multiple approaches to mindfulness conceptualization and related measurement instruments, and present the theoretical foundations that underpin the development of our overarching measurement of mindfulness practice and its beneficial consequences.

### Overview of Mindfulness Theorization and Measurement

A widespread notion of mindfulness defines mindfulness as “the awareness that emerges through paying attention on purpose, in the present moment, and non-judgmentally to the unfolding of experience moment by moment” (Kabat-Zinn, 2003, p. 145). From a theoretical standpoint, the existing literature provides conceptual models, which diversely emphasize mindfulness qualities.

According to Dorjee (2010), a working model for exploring the mechanisms and effects of different types of mindfulness should include the following five dimensions: (1) intention and context of mindfulness practice, (2) bare attention, (3) attentional control, (4) wholesome emotions, and (5) ethical discernment. Moreover, two additional factors that qualify mindfulness practice are (a) meta-awareness (i.e., the function that monitors the meditative process, bringing to awareness distraction or dullness; Wallace, 1999) and (b) insight (i.e., a meta-cognitive understanding of human experience due to a shift in perspective that allows the individual to de-identify with conditioned judgments and direct perception of sensations in the current moment; Shapiro et al., 2006).

An additional model that underpins mindfulness and its measurement has been proposed by Siegel (2018) and is focused on awareness. Specifically, awareness (and, in turn, mindfulness) is defined by a core of six different “facets” as representative of people’s experience of being aware and four additional “spokes” that outline the sources of experience or rather, the things individuals know they can become aware of. The six facets refer to being: (1) receptive, (2) clear, (3) aware, (4) open, (5) peaceful, and (6) calm. To supplement these different streams of awareness, the four spokes are: (a) the first five senses (touch, taste, smell, sight, and hearing), (b) the sixth sense (interior of the body), (c) the seventh sense (mental activities) and (d) the eighth sense (interconnectedness). According to this model, practicing mindfulness is one way for individuals to recognize and accept the stimuli brought into consciousness through one of these senses.

A review of the theoretical conceptualization of mindfulness used to develop eight different renowned self-report questionnaires identified an overall set of nine mindfulness aspects, diversely underpinning each scale development. “The resulting aspects are (1) observing, attending to experiences; (2) acting with awareness; (3) non-judgment, acceptance of experiences; (4) self-acceptance; (5) willingness and readiness to expose oneself to experiences, non-avoidance; (6) non-reactivity to experience; (7) non-identification with own experiences; (8) insightful understanding; and (9) labeling, describing” (Bergomi et al., 2013a, p. 192). Such dimensions also underpin the development of an additional self-report scale, the Comprehensive Inventory of Mindfulness Experiences beta (CHIME-β; Bergomi et al., 2013b).

A converging aspect of mindfulness across different notions is that mindfulness refers to being mentally present in the moment and being fully aware but without any value judgments, worrying or rumination, or rather, being aware and accepting of one’s activities and mental states, as they reveal themselves in the moment (Gunaratana, 1990). In contrast, in their daily life, individuals frequently and spontaneously experience a mindlessness condition understood as the tendency to behave automatically and mechanically (Kabat-Zinn, 1990). In other words, when people perform automatic activities, their mind starts wandering and engages in *distracting* thoughts and feelings that are unrelated to the task at hand and do not remain on a single topic for a long period of time, thus failing to pay attention to and to remember what happens in the surrounding environment (McVay and Kane, 2009). According to the literature (Gunaratana, 1990; Kabat-Zinn, 2020), distractions during mindfulness practice can take the form not only of bodily sensations, memories, sounds, mental formations, restlessness and lethargy, but virtually of any input that assaults our senses and competes for our attention.

Overall, we note that the different aspects and types of stimuli involved in mindfulness and its practice seem to refer to the domains of: (a) cognition (e.g., thoughts, attention, insight, discernment), (b) emotion (e.g., emotions, calm, peace), (c) body/physiology (e.g., five senses, interior of body), (d) physical environment/context (e.g., context of mindfulness practice, situational stimuli), (e) social environment (e.g., interconnectedness), and (f) behavior/action (e.g., non-reaction).

From a measurement standpoint, despite the abundance of factors that conceptually qualify mindfulness and its practice, an overview of the available mindfulness self-report measures seems to suggest that each scale selectively focuses on only some of these aspects. For example, the Mindful Attention Awareness Scale (MAAS; Brown and Ryan, 2003) specifically focuses on the two mindfulness components of awareness and attention, thus targeting only cognition and purposely excluding emotion-related factors such as moods or motivations nor considering contextual or body-related factors. The Five Facet Mindfulness Questionnaire (FFMQ; Baer et al., 2006) captures five factors such as non-reactivity, observing sensations/perceptions/thoughts/feelings, acting with awareness, describing, and non-judging, thus including cognitive, emotional (even though marginally), action-related, contextual and proprioceptive factors but excluding any reference to interrelatedness and the social environment. The Cognitive and Affective Mindfulness Scale-Revised (CAMS-R; Hayes and Feldman, 2004; Feldman et al., 2007) captures the four components of attention, present-focus, awareness, and acceptance and is thus skewed toward cognitive processes. The Freiburg Mindfulness Inventory (FMI; Buchheld et al., 2001; Walach et al., 2006) proposes a molar and mono-dimensional approach to mindfulness understood as a cognitive process of regulation of attention in order to approach experiences with openness, curiosity and awareness. The Langer Mindfulness Scale (LMS; Pirson et al., 2012) focuses on four aspects that include cognitive dimensions (i.e., novelty seeking, novelty producing, flexibility) as well as context-related factors (i.e., engagement, which refers to interacting with the environment). The Kentucky Inventory of Mindfulness Scale (KIMS; Baer et al., 2004) captures the dimensions of observing feelings, thoughts and sensations, describing, acting with awareness and accepting without judgment, thus including cognition-, emotion-, body-, context-, and action-related factors yet excluding the social environment. The Automatic Thoughts Questionnaire (ATQ; Hollon and Kendall, 1980) exclusively focuses on capturing automatic negative thoughts related to depression and, therefore, combines both (although exclusively) emotional and cognitive processes. The Philadelphia Mindfulness Scale (PHLMS; Cardaciotto et al., 2008), only captures the two cognitive factors of awareness and acceptance. Similarly, the State Mindfulness Scale (SMS; Tanay and Bernstein, 2013) relies on a two-component cognition-focused conceptualization of mindfulness tapping attention and orientation to the present (i.e., curiosity, openness and acceptance). The Southampton Mindfulness Questionnaire (SMQ; Chadwick et al., 2008) covers the four aspects of awareness, attention, acceptance and non-reaction, thus representing a mainly cognitive focus. Finally, the Toronto Mindfulness Scale (TMS; Lau et al., 2006) includes both cognitive (i.e., curiosity) and context-related (i.e., decentrating or rather, awareness of surroundings) factors.

To summarize, to date, no mindfulness self-report questionnaire seems to include the measurement of all potential sources of distractions brought to the mind by experience (i.e., cognitive, emotional, bodily/proprioceptive, environmental and social stimuli, behavioral/action). Moreover, no scale is designed to include the measurement of mindfulness aspects experienced by practitioners as a consequence of meditation sessions simultaneously in their general life as well as while at work.

### Mindfulness Practice and Time Flow

The relevance of time for mindfulness and its practice is at least four-fold. First, despite the heterogeneity in mindfulness definitions and theorizations, all conceptualizations converge on the idea that being mindful refers to being “mentally present” in the *present moment* and experiencing a conscious state of mind that focuses on what is experienced in the *present* moment (Gunaratana, 1990; Kabat-Zinn, 2003). For example, the CAMS explicitly provides the “Present focus” dimension that is dedicated to grasp this present-centered component of mindfulness practice. Therefore, time is a framework that is inherently embedded in what mindfulness tries to achieve, and is used to help practitioners make a distinction between present-related stimuli that underpin a mindful state as opposed to past- or future-related stimuli that underpin the distractions of a wandering mind and mindlessness state (Feldman et al., 2007). As such, any mindfulness tool that is structured in a way to help practitioners focus attention on and gain awareness of the timeline of human experience is likely to boost the process of enhanced consciousness and insight.

Second, mindfulness is a function that disarms distractions and could be cultivated through meditation in any moment of our life, even when walking and in motion (Birtwell et al., 2019). However, most formal meditation practices take place in structured sessions (Gunaratana, 1990). We note that mindfulness practice includes an explicit reference to a timeline description of the activities, which points at the difference between preparatory moments and subsequent structured meditation (Kabat-Zinn, 2005). Specifically, a preliminary part of the practice consists in searching and settling the optimal posture which is a key ingredient for the subsequent activities and should not be changed until the practice session is over. Moreover, according to Gunaratana (1990), it is strongly recommended that individuals practice loving-kindness (i.e., an egoless state of awareness achieved by purifying the mind from the damaging psychic irritant arising of resentment) *before* they start their serious practice of meditation. As such, mindfulness practice builds on a time-line flow of activities that unfold in a given time-frame. Therefore, a mindfulness tool that mirrors and operationalizes such a time flow ingredient of the practice may help practitioners to appreciate any gradual change in consciousness throughout different moments and activities of a session.

Third, mindfulness can be considered both a state and a trait (Medvedev et al., 2017), and time is the basic building block to differentiate state mindfulness from trait mindfulness. Briefly, a state is a fluid and short-term mindset and therefore a flexible condition. A trait is a more permanent characteristic of the person and an integral part that is more difficult to change (Geiser et al., 2017). Coherently, state mindfulness refers to a fleeting and ultimately temporary condition in which an individual is aware and able to stay present when distractions arise whereas trait mindfulness is the more stable and permanent ability to enter a mindful perspective at will and over time (Medvedev et al., 2017). For example, the State Mindfulness Scale (Tanay and Bernstein, 2013) is developed to assess self-regulation of attention and awareness relative to one’s immediate experience of a mindfulness session and thus may assist in recognition of one’s single meditation performance. Differently, the Mindful Attention Awareness Scale (Brown and Ryan, 2003) aims at assessing an individual’s general tendency to focus their awareness at will and, therefore, a stable ability to enter a state of mindfulness in their general life.

Fourth, mindfulness meditation is a self-regulation practice that trains attention and awareness in order to bring mental processes under greater voluntary control, thus fostering general mental well-being (Walsh and Shapiro, 2006) in the subsequent moments that follow mindfulness sessions. Consistently, mindfulness scales may help to (a) boost the process of increasing voluntary control of mind over time and (b) strengthen the virtuous circle of mindfulness practice and its beneficial consequences for health. Noteworthy, subsequent moments could be framed in terms of short-term (e.g., right after a session) or long-term (e.g., at periodic points in time after a mindfulness training). For example, the Solloway Mindfulness Survey (Solloway and Fisher, 2007) is designed to track the progress in learning mindfulness practice at a different point in time and specifically asks to rate the consequences of mindfulness practice such as the extent to which mindfulness has taught the individual to stably experience the world in an entirely different way. While existing measures may help in tracking mindfulness progress over time, to our knowledge, no mindfulness scale specifically includes the assessment of mindfulness consequences (e.g., well-being) simultaneously in the short- and long-term period after its practice.

Overall, the review of the literature suggests that time is a dimension inherently involved in mindfulness conceptualization and assessment. However, to date, no existing self-report mindfulness scale engages a timeline approach to both the appraisal of mindfulness experience during a session as well as its consequences after meditation at different points in time.

### Measuring the Experience of Mindfulness Within a Time Framework: The Time Flow Mindfulness Questionnaire

The development of the TFMQ is theoretically grounded in the mindfulness aspects derived from the above review of the literature and developed in order to (a) capture the individual’s experience of mindfulness practice across different temporal windows, ranging from preparatory moments of a session, throughout the practice up to and including the investigation of how the individual feels in the short- and long-term moments following a mindfulness session, (b) assess a comprehensive sample of potentially distracting stimuli from different sources (i.e., *body, cognition, emotion, contextual environment, social environment, and action)* that prevent reaching a mindful state, (c) assess the beneficial consequences of mindfulness practice, and (d) contextualize the mindfulness measurement to both the fields of life in general and work settings. Related to the latter point, given the increasing spreading of mindfulness research and training programs set up for employees in organizational settings (e.g., Google, General Mills; Tan, 2014), a section of the TFMQ is designed to cover the assessment of a mindful state of mind of individuals while at work. Finally, in the current paper the terms body, proprioceptive, and physiological are used interchangeably as they refer to the stimuli that come from inside and outside the body.

The overarching structure of the TFMQ covers three main time windows that respectively focus on what is experienced *before*, *during* and *after* the mindfulness practice. However, the “after” time window is further broken into *short-term*, *long-term* and *long-term at work* moments that follow mindfulness practice. Overall, the TFMQ provides the following time-related item subgroups/sections: (1) preliminary stage (Before Practice), (2) during (During Practice), (3a) right after the practice (Short-Term Benefits), (3b) after the practice in general life (Long-Term Benefits), and (3c) after the practice at work (Benefits at Work). Moreover, *Practice* is a higher order dimension that subsumes the two second order facets of before the practice and during the practice. We use the term “stage” consistent with the mindfulness literature (Gunaratana, 1990) suggesting that every mental state has a birth, a growth and a decay and, therefore, comes in stages. Consistently, individuals should strive to see these stages clearly.

Each time window section provides statements aimed at assessing the individual’s experience of being aware and attentive to the following wide array of potentially distractive types of stimuli: proprioceptive (e.g., bodily sensations, posture), cognitive (e.g., thoughts, fantasies, judgments, memories), emotional (i.e., feelings, emotions), contextual (e.g., inputs from the environment), social/relational (e.g., quality of interactions, listening), and behavioral/action (e.g., suspend reaction).

The TFMQ builds on a conceptualization of mindfulness understood as a set of capacities to achieve a conscious awareness state of mind both during meditation and daily life, which can be actively pursued and/or developed through knowledge and practice (Buchheld et al., 2001; Brown and Ryan, 2004). As such, mindfulness is conceptually half way between a state, in that is changeable and learnable through practice, and a trait that remains relatively stable when individuals are connected with the practice. In other words, the repetition of mindfulness practice can create intentional states of brain activation that may ultimately become traits of the individual (Siegel, 2007). Consistently, the TFMQ is intended for skilled meditators, as well as irregular, casual or novice practitioners. Noteworthy, the completion of the TFMQ requires at least one experience of mindfulness practice.

### Mindfulness and Job Burnout

Job burnout is a work-related stress syndrome caused by prolonged exposure to stressors at work (Maslach, 2003). Burnout is described (Maslach and Leiter, 2008) as the manifestation of emotional exhaustion (i.e., emotional and physical depletion) and cynicism (i.e., a psychological detachment and a negative attitude about one’s work and workplace). An accumulating body of evidence suggests that self-regulation practices that focus on training attention and awareness in order to bring mental processes under greater voluntary control (i.e., mindfulness) contribute to the development of specific capacities such as calmness and concentration, thus fostering general mental well-being (Walsh and Shapiro, 2006). Moreover, mindfulness practice (e.g., MBSR) has been proven to reduce symptoms of perceived work-related stress and job burnout, thus improving employees’ physical and psychological health (Glomb et al., 2011; Hafenbrack, 2017). Specifically, mindfulness is able to change the perception of stressors, rather than acting on the stressors themselves (Hanson and Richardson, 2014; Xiao et al., 2017). As suggested by Siegel (2007), being aware of the present moment (as opposed to experiencing the mind as an amalgam of busy thoughts and feelings, and habitual responses) improves the ability to distinguish different streams of information flow and, therefore, increases the possibility of refraining from self-defeating thought-patterns and maladaptive patterns of emotional reactivity thus reducing mental suffering (Luken and Sammons, 2016). As such, we expect that mindfulness practice may exert it’s benefits on employees’ functioning at work by enhancing their ability to detect when a distraction is potentially interfering with both the performance of a work task (Chaskalson, 2011) and attentive listening and relating with other people at work (Langer and Moldoveanu, 2000). In turn, the beneficial consequences of mindfulness experienced by employees may contribute to prevent them from experiencing feelings of exhaustion and detachment from their work (i.e., burnout). Consistent with the above arguments and prior empirical results, we expect to find:

*Hypothesis 1*: The “practice” dimension of the TFMQ (i.e., preliminary, during) will negatively predict emotional exhaustion and cynicism, both directly and indirectly through the “benefits at work” dimension of the TFMQ.

## Study 1: Construct Validity

### Method

#### Participants and Procedure

The sample consisted of *N* = 141 individuals from the general population who experienced mindfulness practice. Sixty-eight percent of respondents were female. The mean age of participants was 44.73 years (*SD* = 11.8). The time of experience with mindfulness practice ranged from less than a year to 37 years with a mean of 7.6 years (*SD* = 6.9). Twenty-seven percent of respondents had a previous experience with meditation practice, 3% with yoga, while the remaining 70% indicated both meditation and yoga. Overall, the sample size was appropriate to perform our statistical analyses in that all Exploratory Factor Analyses satisfied a subject to item ratio higher than 5:1 and up to 10:1 (Hatcher, 1994).

The research staff provided participants with informed consent materials that explained the anonymous nature of the data collection and their rights as research participants, and distributed the questionnaire in a sealed envelope in order to assure confidentiality. Participants were recruited during their participation in mindfulness courses or training, and either completed the survey containing the research measures after their training or completed the questionnaire at home.

#### Measures

Below is a description of the measures used in the data collection for the current analyses.

##### Time Flow Mindfulness Questionnaire

The TFMQ is a 42-item self-report instrument developed in order to assess the cognitive, emotional, bodily, context-related (environmental, social), and behavioral/action inputs experienced by the mind at three different times (stages) of the mindfulness practice (see Appendix 1): (1) preliminary stage (Before Practice), a sample item from this 8-item subscale is, “*Before starting the breathing/meditation, I feel comfortable when I take the position*”; (2) during stage (During Practice), a sample item from this 10-item subscale is, “*During the activities of visualization/concentration on the present, I do not get carried away by any emotions I feel”;* (3a) right after the practice (Short-Term Benefits), a sample item from this 10-item subscale is, *“Right after finishing visualization/concentration activities on the present, I perceive a general sense of well-being”;* (3b) after the practice in general life (Long-Term Benefits), a sample item from this 15-item subscale is, *“Thanks to the activities of visualization/concentration on the present*, *I am more aware of what happens around me moment by moment”*; (3c) after the practice at work (Benefits at Work), a sample item from this 15-item subscale is, *“Thanks to the activities of visualization/concentration on the present, in difficult situations at work I can suspend my reactions and not act immediately.”* Each block of items related to the five different time-related sections (1, 2, 3a, 3b, 3c) provides a specific lead-in that instructs respondents to relate statements to different time frames. The items were newly formulated by the authors, two of whom have personal experience with mindfulness meditation and Buddhist psychology, in order to cover the above different types of potential distractors across the three different time windows and assess the beneficial consequences of mindfulness. Items were rated on a 5-point frequency scale ranging from 1 (*Never)* to 5 (*Always*). Items were worded both positively and negatively in order to avoid response set. Negative items were reversed such that higher scores reflect greater levels of positive experiences associated to mindfulness.

#### Analytical Strategy

We first performed Exploratory Factor Analyses (EFA) on the TFMQ items using SPSS 2.0, in order to assess the dimensionality of the TFMQ. For the EFA analyses, oblique rotation methods were used and decisions concerning the number of factors to extract were based on scree plots of eigenvalues and the hypothesized theoretical structure of the TFMQ. Next, we tested Confirmatory Factor Analysis (CFA) models using the Maximum Likelihood Robust estimator, by the means of MPlus 8.0 (Muthén and Muthén, 1998-2017). In order to examine model fit, we used the following goodness-of-fit indices, as recommended by the literature (Byrne, 2006; Meade et al., 2008): the Root Mean Square Error of Approximation (RMSEA), the Comparative Fit Index (CFI) and the Tucker-Lewis index (TLI). RMSEA is considered an absolute fit index that estimates lack of model fit and compensates for model complexity, with values of 0.05 or lower indicating a well-fitting model, 0.05–0.08 indicates a moderate fit, and 0.10 or greater indicates poor fit (Browne and Cudeck, 1993). The CFI and TLI are considered incremental fit indices that compare the model of interest with a null or independence model (Bentler, 1990), with values of 0.90–0.95 indicating acceptable fit and values above 0.95 indicating good fit (Hu and Bentler, 1999). Finally, Cronbach’s alpha was performed to assess the internal consistency of TFMQ facet scales. Subsequently, items that resulted to be indicators of a latent variable were then used to compute the related mean score of the TFMQ sub-dimensions.

It should be emphasized that analyses on the TFMQ subscales (before practice, during practice, short-term benefits, long-term benefits, benefits at work) were performed separately in that they include the assessment of proprioceptive, cognitive, emotional and context-related inputs experienced by the mind, yet repeated across different time frames (before, during, after) of mindfulness. Additionally, the proprioceptive, cognitive, emotional and context-related dimensions are repeatedly assessed both in the realm of general life (long-term benefits) as well as in the specific domain of work settings (benefits at work). Finally, consistent with Hatcher’s (1994) recommendations, all EFAs were performed by following a subject to item ratio higher than 5:1 and up to 10:1.

### Results

#### Psychometric Properties of the Time Flow Mindfulness Questionnaire

The EFA on the eight items of the before practice subscale extracted three factors that provided the best solution, and explained 71.6% of the total variance. Factor 1 was defined by the items referring to inputs from the body while taking the position, Factor 2 was defined by items referring to breath, while Factor 3 was loaded by items referring to thought and emotions crossing the mind in the present moment. The EFA on the 10 items of the during practice subscale extracted two factors. This solution accounted for 56.6% of the cumulative variance, with most of this accounted for by Factor 1 (40.1%), which was a blend of all of the bodily, breath, cognitive, emotional, and environmental inputs. Since Factor 2 was defined by the only two negative items of this subscale, we interpreted this as a method factor and settled for a final mono-factorial structure of this subscale. The EFA on the four items of the short-term benefits subscale extracted two factors that provided the best solution and explained 85.8% of the total variance. Factor 1 was defined by the items referring to body and general well-being, and Factor 2 was defined by items referring to cognitions and emotions. The EFA on the 15 items of the long-term benefits subscale extracted four factors that provided the best solution and explained approximately 70% of the total variance. Factor 1 was defined by the items referring to positive cognitions and emotions, Factor 2 was defined by items referring to positive relational experiences (i.e., social context), Factor 3 was loaded by items referring to attention to the environment, while Factor 4 was loaded by items referring to awareness of body and posture. Finally, the EFA on the five items of the benefits at work subscale extracted two factors that provided the best solution and explained 79.8% of the total variance. Factor 1 was defined by the items referring to positive relational experiences, while Factor 2 was loaded by items referring to awareness of body and posture.

Table 1 shows the results from the CFAs on the before practice, during practice, short-term benefits, long-term benefits and benefits at work subscales. Additionally, we tested a CFA on an overall Practice scale including the preliminary and during subscales, and a Benefits Total scale including short-term and long-term benefits subscales. As can be seen, all CFAs showed excellent fit indices with RMSEAs ranging from 0.000 to 0.066, CFIs ranging from 0.955 to 1.0, and TLIs ranging from 0.943 to 1.0. Overall, these results demonstrated the appropriateness of the hypothesized latent factors for each of the TFMQ subscales.

**TABLE 1:** Results of tests for CFA.

		Model Fit					
	Models(M)	χ^2^	*df*	RMSEA (90% CI)	CFI	TLI	α
PRACTICE	M1: Preliminary (3-factor) M2: During (2-factor) M3: PRACTICE TOTAL (5-factor)	17.242 48.244 156.837	17 34 125	0.010 (0.000–0.077) 0.055 (0.000–0.088) 0.043 (0.015–0.062)	0.999 0.981 0.964	0.999 0.978 0.956	0.73; 0.89; 0.83 [0.76] [0.82] [0.88]
BENEFITS	M4: Short-term Benefits (2-factor) M5: Long-term Benefits (4-factor) M6: BENEFITS TOTAL (6-factor) M7: Benefits at Work (2-factor)	0.001 135.053 194.139 5.138	1 84 137 4	0.000 (0.000-0.000) 0.066 (0.044-0.086) 0.054 (0.035-0.071) 0.048 (0.000-0.152)	1.00 0.955 0.960 0.994	1.04 0.943 0.950 0.985	0.82; 0.59 [0.81] 0.81; 0.83; 0.92; 0.87 [0.93] [0.93] 0.81; 0.84 [0.85]

*RMSEA, Root Mean-Square Error of Approximation; CFI, Comparative Fit Index; TLI, Tucker-Lewis Index. Alpha coefficients in square brackets refer to the whole scale.*

Overall, the Cronbach’s alpha for the empirical scales ranged from 0.73 to 0.93 (see Table 1), with the only exception of a 0.59 alpha for one factor of the short-term benefits subscale, which nevertheless shows an overall 0.81 alpha. Together, these results on the psychometric properties of the TFMQ provide support for its construct validity.

## Study 2: Concurrent Validity

### Method

#### Participants and Procedure

Study 2 was conducted to provide data on the concurrent validity of the TFMQ. Specifically, we explored correlations between the TFMQ scores and the Five Facets Mindfulness Questionnaire, which measures multiple mindfulness dimensions and, therefore, diverse sources of stimuli. The reason for using the FFMQ is both conceptual and practical in nature. From a theoretical standpoint, the FFMQ is a measure of mindfulness built on a comprehensive review of existing scales which provides an extensive coverage of most mindfulness dimensions and distractive stimuli. From a practical standpoint, the FFMQ is a renowned and one of the most utilized mindfulness measure already validated and available in Italy.

We collected anonymous paper and pencil survey data on a sample of Italian participants to five different MBSR courses. Data were collected at two time points: baseline during the 4th week of the MBSR course (Time 1), and a 1-month follow-up during the 8th week of the MBSR (Time 2). The initial sample consisted of *N* = 65 individuals at Time 1. Of these, *N* = 58 completed the second survey, resulting in a 89% average retention rate. Seventy-four percent of respondents were female. The mean age of participants was 44.9 years (*SD* = 12.3). About 75% had a college, or higher, degree, while 25.5% were high school graduates. Forty-five percent of respondents had a previous experience with meditation practice, 5.9% with yoga, while the remaining 49% indicated other unspecified experiences. We conducted a power analysis to determine the minimum sample size to detect a moderate positive correlation among each couple of study variables of interest. After setting a one-tailed level of α = 0.025 and 1–β = 0.80, results suggested that the minimum sample size required was respectfully of 46 and 29 subjects to detect an effect size of ρ = 0.40 and ρ = 0.50.

Participation was voluntary and not rewarded by any incentive. In order to assure anonymity, while collecting the longitudinal data, participants were assigned a code. Members of the research team provided participants with informed consent materials that explained the anonymous nature of the data collection and their rights as research participants, and distributed the questionnaire. Respondents were allowed to complete the survey at home and return it in a sealed envelope to the research team, in order to assure confidentiality.

#### Measures

Below is a description of the measures used in the data collection for the current analyses.

##### Time Flow Mindfulness Questionnaire

The scale is the same as previously described in Study 1. For the purpose of the current study, we computed the following TFMQ dimensions: preliminary (8 items), during (10 items), benefits (19 items), and benefits at work (5 items).

##### Five Facets Mindfulness Questionnaire

We used the Italian version (Giovannini et al., 2014) of the Five Facets Mindfulness Questionnaire (FFMQ; Baer et al., 2006). The FFMQ is a 39-item self-report instrument developed to measure one general mindfulness factor and five secondary facets: (1) Observe (eight items), which refers to attending to sensory stimuli that mainly derive from external sources and the body as well as related cognitions and emotions; (2) Describe (eight items) taps labeling internal experiences with words; (3) Act with Awareness (eight items), an ongoing attention to and awareness of present activity and experience; (4) Non-judge (eight items), having a non-evaluative attitude toward one’s thoughts and emotional processes while focusing on inner experiences, rather than taking on a critical stance; and (5) Non-react (seven items), assuming a stance that implies being able to perceive thoughts and feelings, especially when they are distressing, without feeling compelled to react or being overwhelmed. Items were assessed on a 5-point Likert scale, ranging from 1 (never or very rarely true) to 5 (very often or always true), with higher total scores reflecting a greater degree of mindfulness.

#### Analytical Strategy

In order to assess the concurrent validity of the TFMQ, we examined the 1-month time-lagged correlations of the TFMQ dimensions (preliminary, during, benefits, benefits at work) with the five FFMQ facets (observe, describe, act with awareness, non-judge, non-react) first at Time 1 (4th week of the MBSRs), and then at Time 2 (8th week of the MBSRs).

### Results

#### Descriptive Statistics, Reliabilities and Correlations

Table 2 presents the descriptive statistics, scale reliabilities, and intercorrelations among the study variables at Time 1 (T1) and Time 2 (T2). As shown in the diagonal of Table 2, each study variable met the criterion for internal consistency reliability, ranging from 0.75 to 0.93. Intercorrelations at T1 show that the preliminary dimension of the TFMQ positively correlates only with the *non-react* facet of the FFMQ, while the during dimension positively correlates with observe, act with awareness, non-react, non-judge, but not with describe. Interestingly, mindfulness benefits of the TFMQ positively correlates with all five FFMQ facets, whereas benefits at work positively correlates only with observe, describe, and act with awareness. When considering the correlations at T2, results show an interesting pattern of relationships. Overall, the number of significant correlations at T2 increased in comparison to T1. Specifically, the preliminary dimension of the TFMQ positively correlates with both non-react and act with awareness. Moreover, each of the dimensions during, benefits and benefits at work positively correlates with all five FFMQ facets.

**TABLE 2:** Descriptive statistics, correlations, and Cronbach’s alphas.

Variable	Mean	*SD*	1	2	3	4	5	6	7	8	9
(1) FFMQ – *Observe*	3.6 (3.9)	0.62 (0.62)	[0.75]	0.37**	0.38**	0.29*	0.49**	0.08	0.50**	0.53**	0.45**
(2) FFMQ – *Describe*	3.9 (4.0)	0.63 (74)	0.40**	[0.87]	0.41**	0.38**	0.33*	–0.13	0.35**	0.36**	0.44**
(3) FFMQ – *Act with Awareness*	3.2 (3.6)	0.82 (0.73)	0.29*	0.35**	[0.93]	0.62**	0.46**	0.30*	0.49**	0.48**	0.55**
(4) FFMQ – *Non-judge*	3.7 (3.9)	0.87 (0.80)	0.24	0.33*	0.63**	[0.90]	0.42**	–0.05	0.42**	0.44**	0.44**
(5) FFMQ – *Non-react*	3.2 (3.4)	0.60 (0.56)	0.21	0.13	0.61**	0.54**	[0.84]	0.26*	0.58**	0.52**	0.53**
(6) TFMQ – *Preliminary*	3.6 (3.9)	0.58 (0.51)	0.09	–0.02	0.21	0.11	0.34*	[0.80]	0.35**	0.31*	0.27
(7) TFMQ – *During*	3.5 (3.8)	0.52 (0.50)	0.29*	0.25	47**	0.39*	0.44**	0.34**	[0.79]	0.58**	0.45**
(8) TFMQ - *Benefits*	3.4 (3.7)	0.60.57)	0.46**	0.47**	0.51**	0.41**	0.51**	0.32*	0.69**	[0.92]	0.81**
9. TFMQ - *Benefits at Work*	3.3 (3.6)	0.84 (74)	0.66**	0.40**	0.36*	0.19	0.26	0.26	0.37*	0.70**	[0.88]

*p < 0.05; **p < 0.01; ***p < 0.001. Numbers outside the brackets refer to the Time 1 and numbers in brackets refer to the Time 2. Correlations below the diagonal are for the Time 1 and correlations above the diagonal are for the Time 2. Cronbach’s alpha reliabilities are in square brackets in the diagonal.

## Study 3: Predictive and Discriminant Validity

### Method

#### Participants and Procedure

The sample and procedure were the same as presented in Study 1. In terms of organizational variables, 77% of respondents worked as full-time workers and 75.8% were permanent workers. Approximately 47% were white-collar workers, 21% were managers or supervisors, 2.1% were blue-collars, and the remaining 30.9% did not specify. We used a lower bound approach (Westland, 2010) to determine the minimum sample size in relation to the conceptual SEM illustrated below to obtain medium to large values (respectively, δ = 0.35 to δ = 0.50) of the expected minimum correlations among the first-order latent variables of the model. After setting a one-tailed level of α = 0.05 and 1–β = 0.80, results suggested that the minimum sample size to detect the above effects ranged between 100 (for δ = 0.35) and 110 (for δ = 0.50) subjects.

#### Measures

##### Time Flow Mindfulness Questionnaire

For the purpose of the current study, we used the preliminary (8 items) and during (10 items) facets of the TFMQ, which were collapsed in an overall Practice (18 items) dimension, and the Benefits at Work (5 items) subscale. The scales are the same as previously described in Study 1.

##### Job Burnout

The Italian version (Borgogni et al., 2005) of the Maslach Burnout Inventory—General Survey (Schaufeli et al., 1996) was used, including five items measuring exhaustion and six items assessing cynicism. A sample exhaustion item is, “*I feel emotionally drained from my work*” and a sample cynicism item is, “*I doubt the significance of my work.*” Items were rated on a 7-point frequency scale ranging from *never* (0) to *daily* (6).

##### Control Variables

As recommended by Spector and Brannick (2011), a measure of social desirability bias was included in the survey to control for common method variance. A three-item self-report measure of Social Desirability (Petitta and Di Cave, 2011) was used to evaluate the tendency to answer questions in a manner that one expects to be viewed favorably by others. Respondents were asked to indicate their agreement with the statements using a 7-point Likert scale ranging from 1 (*Totally disagree*) to 7 (*Totally agree*). A sample item is, “*I immediately appear delightful to everyone.”*

#### Analytical Strategy

**FIGURE 1:**
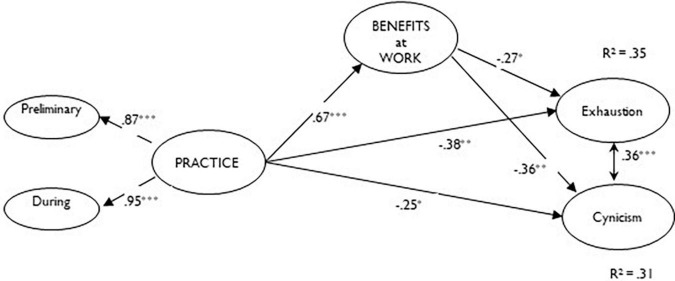
Standardized coefficients for the final structural model. ^***^p < 0.001, ^**^p < 0.01, *p < 0.05.

In order to maximize the reliability and parsimony of our SEM and to reduce sources of sampling error (Little, 2013), the initial pool of items for the practice and benefits at work dimensions of the TFMQ were grouped into three parcels. Following Little et al. (2013) recommendations, items were sequentially assigned to parcels based on their item-total corrected correlation. In order to initially assess the distinctiveness among the study constructs, we tested a six-factor CFA model in which: (a) the items that referred to the preliminary and during TFMQ sub-dimensions loaded onto a unidimensional second-order practice factor; and (b) each item that referred to benefits at work, exhaustion, cynicism, and social desirability loaded onto the additional five unique latent factors. Next, we tested a SEM wherein mindfulness practice predicts exhaustion and cynicism, both directly and indirectly through mindfulness benefits at work, and controlling for social desirability (see Figure 1 for a nomological network). All models were tested on the covariance matrix, using the weighted least squares—mean and variance adjusted (WLSMV) estimator with MPlus 8.0 (Muthén and Muthén, 1998-2017).

### Results

#### Descriptive Statistics and Correlations

Means, standard deviations, alpha coefficients and zero-order correlations among the scales are reported in Table 3. Overall, Cronbach’s alpha of empirical dimensions ranged from 0.81 to 0.92.

**TABLE 3:** Descriptive statistics, correlations, and Cronbach’s alphas.

Variable	Mean	*SD*	1	2	3	4	5
(1) Practice	3.9	0.51	(0.88)				
(2) Benefits at Work	3.6	0.76	0.52**	(0.85)			
(3) Exhaustion	2.0	1.35	−0.44**	−0.46**	(0.92)		
(4) Cynicism	1.7	1.38	−0.30**	−0.36**	0.49**	(0.81)	
(5) Social Desirability	4.7	1.02	0.32**	0.29**	–0.15	−0.35**	(0.79)

Cronbach’s alpha reliabilities are in brackets on the diagonal; **p < 0.01.

#### Measurement Model

Results from the six-factor CFA showed good fit indices: χ^2^(241, *N* = 141) = 391.871, *p* < 0.001, RMSEA = 0.067 (0.054;0.078), CFI = 0.94, TLI = 0.93, thus supporting the appropriateness of the six hypothesized latent factors and the distinctiveness among the study variables. As such, the TFMQ dimensions that assess the benefits of mindfulness showed discriminant validity from other well-being measures.

#### Structural Model

The mediation model examining mindfulness practice and benefits at work as predictors of job burnout (exhaustion, cynicism) showed an adequate fit to the data: χ^2^_(df = 243, *N* = 141)_ = 421.701, *p* < 0.001, RMSEA = 0.072 (0.061 –0.084), CFI = 0.93, TLI = 0.92. Specifically, while χ^2^ resulted significant, all of the remaining fit indices showed values above the recommended cut-off thresholds (Hu and Bentler, 1999). Moreover, our results are in line with previous literature (Muthèn and Kaplan, 1985; Chou et al., 1991) suggesting that small or moderate sample sizes, as is the case of the current study, may inflate Type I error rates and affect χ^2^ significance, thus running the risk that models which provide acceptable fit to the data according to other types of fit indices (as is the case of CFI, TLI, and RMSEA in our model) are at a risk of being erroneously judged to be a poor fit. As can be seen (Figure 1), mindfulness practice positively predicted benefits at work (0.67, *p* < 0.001) and negatively predicted both exhaustion (–0.38, *p* < 0.01) and cynicism (–0.25, *p* < 0.01). Similarly, mindfulness benefits at work exerted negative effects on both exhaustion (–0.27, *p* < 0.05) and cynicism (–0.36, *p* < 0.01). Finally, mindfulness practice exerted a negative indirect effect on both exhaustion (–0.18, *p* < 0.05) and cynicism (–0.24, *p* < 0.01). The results were achieved while controlling for social desirability, which was negatively associated to cynicism (–0.58; *p* < 0.001) but not to exhaustion. Overall, the model explained 31% of the cynicism variance and 35% of the exhaustion variance.

### Discussion

The primary purpose of the current paper was to examine the psychometric properties of the TFMQ, a new self-report mindfulness scale designed to (a) measure cognitive, emotional, bodily, contextual (environmental, social), and action-related inputs experienced by the mind across three different time windows of the mindfulness practice (before, during, and short-term as well as long-term after moments), (b) assess the beneficial consequences of mindfulness practice (i.e., a mindful condition after meditation), and (c) contextualize the mindfulness measurement to both the fields of life in general and work-life. Toward this end, three studies were conducted: the first aimed to examine the factorial structure and internal consistency of the TFMQ; the second aimed to assess its convergent validity through correlations with other mindfulness measures (i.e., FFMQ facets); and the third aimed to examine its discriminant and criterion validity by testing a nomological network that posits mindfulness practice (i.e., a TFMQ dimension) as a predictor of mindfulness benefits at work (i.e., a TFMQ dimension), which in turn negatively predict exhaustion and cynicism (i.e., job burnout) in a sample of practitioners who held a job.

Overall, our findings suggest that the TFMQ and its dimensions showed the expected patterns in construct, convergent, discriminant and criterion validity analyses, as well as good internal consistency of empirical factors. Specifically, in a sample of both regular as well as casual meditators, results from EFAs and CFAs on the TFMQ items suggest that all items loaded on the expected latent factors. The before practice dimension included three factors that respectfully tap into bodily/proprioceptive, breath, and cognitive and emotional distracting stimuli experienced by individuals in the preliminary moments when preparing for meditation. All items of the during practice dimension loaded onto one single factor that included a blend of bodily/proprioceptive, breath, cognitive, emotional and environmental stimuli detected by individuals during the structured meditation. The short-term after the practice (benefits) dimension included two factors that respectfully tap into bodily and general well-being sensations, as well as cognitive and emotional inputs experienced by individuals right after a meditation session. Next, the long-term after the practice (benefits) dimension included four factors that respectfully tap into cognitive and emotional, relational (social environment), contextual (physical environment), and bodily inputs experienced by individuals in their general life long after the meditation sessions. Finally, the “long-term after the practice at work” (benefits at work) dimension included two factors that tap into bodily as well as relational and non-reactive sensations experienced by individuals (who held a job) in their work life and long after the meditation sessions. Moreover, the TFMQ factors across all three time windows (before, during, after) captured the individual’s mindful orientation to experience reality in an attentive, aware, observing, present-focus, accepting, non-reactive, acting with awareness and interconnected way. As such, the TFMQ provides incremental value over the existing scales reviewed in our introduction, which all selectively include only a combination of some of these mindfulness components (e.g., Bergomi et al., 2013a,b; Ackerman, 2020).

Moving further on, results from the second two-wave study on trainees of MBSR courses suggest that the TFMQ dimensions (preliminary, during, benefits and benefits at work) correlate with all five FFMQ dimensions at Time 1 (4th week of course) and display an increased pattern of correlations at Time 2 (the end of the MBSR course). As such, our findings provide support for the convergent validity of the TFMQ with a comprehensive mindfulness measure (i.e., FFMQ) that captures up to five different and widely acknowledged aspects of mindfulness (i.e., non-reactivity, observing sensations/thoughts/feelings, acting with awareness, describing, and non-judging) and, therefore, a comprehensive array of distracting stimuli (i.e., cognitive, emotional, action-related, contextual and proprioceptive; yet, not social). On the one hand, we note that an increased pattern of correlations at Time 2 is consistent with our conceptualization of mindfulness understood as a set of capacities to achieve a conscious awareness state of mind both during meditation and subsequently during daily life, which can be actively pursued and/or developed through knowledge and practice (Brown and Ryan, 2004). As such, the mindful state of mind captured by the TFMQ is both a state of the individual that repeated practice can turn into an intentional state of brain activation that may ultimately become a trait of the individual (Siegel, 2007). On the other hand, the TFMQ dimensions also include the additional “relatedness/interconnectedness” aspect of mindfulness (i.e., social environment) not included in the FFMQ, thus showing an incremental value over other renowned and widely used comprehensive mindfulness measures.

Noteworthy, results from the third study on the effects of the TFMQ dimensions on job burnout conducted among practitioners (who held a job) support both the discriminant as well as the criterion validity of the new scale. Not only did the TFMQ dimensions (i.e., practice, benefits at work) prove to be empirically distinct from other health-related constructs (i.e., burnout), but they also were found to significantly contribute to explaining the variance in workers’ feelings of exhaustion and psychological detachment from their work. Specifically, higher levels of a mindful state experienced during the practice were associated to lower levels of job burnout. Similarly, higher levels of a mindful state experienced by practitioners as a consequence of mindfulness sessions and while at work, was associated to lower burnout. Moreover, while practice seemed to exert higher effects in preventing workers’ emotional exhaustion, beneficial consequences of mindfulness practice at work (i.e., benefits at work) exerted higher effects in preventing workers’ detachment from work. This is likely because the benefits at work dimension also includes a relational aspect and grasps the extent to which mindfulness practice enables practitioners/workers to experience an increase in conscious and attentive listening to what other employees say and, therefore, more positive social interactions at work (Hanson and Richardson, 2014). As such, it encourages the prevention of callous feelings of detachment and distance from one’s job that substantiates the cynicism facet of burnout (Maslach and Leiter, 2016). By disentangling the measurement of a mindful state experienced by individuals during the practice from the one experienced long after the practice in their general life and at work, the TFMQ enables us to unfold the role of both the practice itself as well as it’s beneficial consequences (i.e., a sense of well-being and a mindful state that underpins wise action during life after meditation sessions). Overall, our findings have implications for the existing literature in the area of occupational health by contextualizing the operationalization of mindfulness as well as its benefits to organizational contexts, thus contributing to further expand the study of the link between mindfulness practice and well-being at work.

The key role of mindfulness practice and its benefits at work in preventing work-related stress as assessed by the TFMQ appears to be particularly interesting during the current spread of the Coronavirus disease (COVID-19). Specifically, the pandemic has brought to the forefront the relevance of protecting workers from the psychological effects of emotional pressure shared among people working under stressful situations (e.g., medical emergency; Brennan and Oeppen, 2020). Organizations are advised to develop an emotional ecosystem that can foster healthy and safe work conditions, whether being performed remotely or in physical presence or blended, and create an environment of employee mindful self-care that emphasizes the significance of attending to the development of one’s own inner life in order to do their job well (Siegel, 2010), which likely cascades and spills over to coworkers generating positive spirals (Singh et al., 2021).

The findings of our study are of practical relevance from several standpoints. First, mindfulness is to be considered a conscious state of mind that any human being might potentially achieve (Kabat-Zinn, 2005). As such, people can enter a state of mindfulness through specific practice proposed by mindfulness training that involves the cultivation of a complex set of cognitive operations that tend to counteract mind-wandering and, therefore, can be challenging to learn or apply. The goal of the practice is to become fully aware of all facets of one’s experience and to bring mental processes under greater voluntary control by training attention to recognize and accept the stimuli brought into consciousness. Given the wide-ranging types of potentially distracting stimuli operationalized by the TFMQ, this new scale may help both skilled as well as naïve practitioners to increase their awareness of different types of inputs potentially undermining the mind in the present. Noteworthy, this may help individuals in gaining awareness of idiosyncratic tendencies in mind wandering on specific types of stimuli (e.g., emotional) as compared to the many other sources of distractions potentially active. For example, an individual may notice that the most recurrent source of distractions during their meditation practice is emotional agitation or, alternatively, imperfections in the posture and misalignments in one’s sitting or, alternatively, being sidetracked by random thoughts and their association. Reading the TFMQ items after a mindfulness session is a form of facilitation of the process of increasing awareness of the self and could be used both as a structured activity in formal training with mindfulness trainees as well as a spontaneous activity carried informally by any mindfulness practitioner who wishes to evolve in their ability to meditate.

Second, carrying over one’s meditation into the events of daily life is not a simple process and the transition point between the end of a meditation session and the beginning of “real life” is a long jump because the achieved calm and concentration can evaporate within minutes (Gunaratana, 1990). The TFMQ may assist in strengthening the effects of mindfulness practice by allowing trainees to more clearly associate what they experience during their mindfulness practice to how they feel during their general life as a consequence of their mindfulness training. To this end, structuring the TFMQ into sections purposely designed to assess the beneficial consequences of mindfulness practice after the sessions and also later in general life may help to track a practitioners’ and/or a naïve trainee’s progress through a long-term attempt to become more mindful and enhance concentration. Specifically, given the TFMQ split between evaluation of “short-term” and “long-term” mindfulness benefits, the new tool may assist individuals in gaining awareness on their progress toward the ability to reach voluntary control of the mind at will and whether this state of mind extends over time or, conversely, is still short-lasting and deserves further training.

Third, meditation is a psychological activity and is very sensitive to the attitude with which individuals are motivated to deal with the raw stuff of their magmatic mind and, therefore, how they approach each session (Gunaratana, 1990). As such, the first obstacle to increase mindfulness is one’s own intention and motivation to meditate (Kabat-Zinn, 2005). This may be particularly relevant when attempting to implement mindfulness practice in the specific domain of life at the workplace where employees happen to be the clients of mindfulness programs that are set by the employer. While there is initial evidence of existing training programs set up for employees in organizational settings (e.g., Google, General Mills; Tan, 2014), each organization has to deal with the challenge of successfully involving employees in mindfulness practice and motivating them to attend a training. Here, the TFMQ may assist in at least two ways. On the one hand, the fine-grained assessment of multiple types of distracting stimuli may assist in the development of mental skills such as explicitly paying attention to different types of inputs, and therefore facilitate the meditation “task” particularly for employees that are naïve meditators/trainees toward increasing their awareness. As such, the new tool may facilitate naïve employees in appreciating any gradual change in consciousness throughout different moments and activities of a session, thus increasing a sense of mastering of the practice. On the other hand, the benefits at work section may provide employees with a self-assessment tool that further bridges meditation practice with its positive effects on attentive and productive carrying out of work activities as well as profitable relationships with others. Finally, at the organizational level, since the TFMQ may be used to assess how the mindfulness practice and the benefits of mindfulness at work predicts the well-being of employees (i.e., lower levels of job burnout), organizations may track the welfare of their context and monitor the advantages of interventions aimed at implementing mindfulness training programs.

## Limitations, Future Directions, and Conclusion

While the three studies make several contributions to the extant literature, they also suffer from some limitations that should be addressed in future research efforts. First, our research only included self-report data from convenience samples and we did not collect data from multiple sources (e.g., supervisor’s assessment of worker’s burnout) and/or use multiple methods (e.g., objective physiological measure of the trainee’s health status, such as cortisol levels). Therefore, it is unclear if self-selection biases in the kind of respondents that agreed to participate may have affected our findings. Second, an additional limitation is the cross-sectional nature of the data of Study 3, which does not allow us to draw causal conclusions on the hypothesized conceptual model. Future studies gathering cross-lagged data could better delineate the causal effects of mindfulness practice and subsequent benefits on trainees’ well-being in their general life, and at work in particular. A third arguable limitation is the size of our samples. However, we note that the sample size of Study 1 is consistent with Hatcher’s (1994) recommendations of a subject to item ratio of 10:1 in EFA and with previous validation studies of mindfulness scales (e.g., CHIME-β; Bergomi et al., 2013b). Moreover, not only Study 2’s sample consisted of participants from five different MBSR courses, but the simultaneous existence of high and low correlations among the study variables suggests that common method bias unlikely occurred in our study (Spector, 2006). Nonetheless, additional replication of our results on larger samples would be useful. Finally, future studies should examine the ecological validity of the TFMQ and verify that our results on Italian samples were not context dependent (i.e., to determine if they would generalize to a different cultural context), by replicating (and extending) our findings to different languages and cultural settings.

## Data Availability Statement

The datasets presented in this article are not readily available because we do not have permission from participants to share data. Requests to access the datasets should be directed to LP, laura.petitta@uniroma1.it.

## Ethics Statement

Ethical review and approval was not required for the study on human participants in accordance with the local legislation and institutional requirements. The patients/participants provided their written informed consent to participate in this study.

## Author Contributions

LP designed and executed the studies, developed the questionnaire, analyzed the data, and wrote the manuscript. ES contributed to develop the questionnaire, collected the data, and collaborated in writing the section “Introduction.” MG contributed to develop the questionnaire, assisted in collecting the data, and collaborated in writing some parts of the section “Introduction.” MP contributed to develop the questionnaire and assisted in collecting the data. All authors approved the final version of the manuscript for submission.

## Conflict of Interest

The authors declare that the research was conducted in the absence of any commercial or financial relationships that could be construed as a potential conflict of interest.

## Publisher’s Note

All claims expressed in this article are solely those of the authors and do not necessarily represent those of their affiliated organizations, or those of the publisher, the editors and the reviewers. Any product that may be evaluated in this article, or claim that may be made by its manufacturer, is not guaranteed or endorsed by the publisher.

## Appendix

**APPENDIX 1:** Time Flow Mindfulness Questionnaire scale items.

*Practice*
**Preliminary**
Before starting the breathing/meditation … (1)… *I feel comfortable when I take the position* (2)… *I feel relaxed when I take the position* (3)… *I feel tense when I take the position (–)* (4)… *I feel that some part of my body bothers me (–)* (5)… *I think of so many things that I have to strain to focus on right now (–)* (6)… *I’m so taken to my emotions that I have to strain to focus on right now (–)*
When paying attention to my breath I am so … (7)… *taken in my thoughts that I cannot follow any instructions (–)* (8)… *involved in my emotions that I cannot follow any instructions (–)*
**During**
During the activities of visualization/concentration on the present… (9)… *I notice that my breathing becomes fuller and deeper* (10)… *I am not distracted from the surrounding environment* (11)… *I accept the feelings that come from my body and experience them as they are* (12)… *if I get distracted, I can easily refocus my attention on the here and now* (13)… *I do not get distracted by the flow of my thoughts* (14)… *I am aware of any emotions that arise in me* (15)… *I do not get carried away by any emotions I feel* (16)… *if I get distracted, I can no longer return to the here and now (–)* (17)… *if I’m distracted by emotions that I feel, I can no longer return to the here and now (–)* (18)… *if I get distracted, I can accept it serenely and do not judge myself severely*
***Benefits***
**Short-term benefits**
Right after finishing visualization/concentration activities on the present… (19)… *I perceive a general sense of well-being* (20)… *I feel my body more alive* (21)… *my mind feels quieter* (22)… *my mind is once again filled with thoughts/emotions (–)*
**Long-term benefits**
Thanks to the activities of visualization/concentration on the present… (23)… *I feel my body less stiff and tense* (24)… *I am more aware of my posture* (25)… *if I have a negative thought, I know how to recognize it, accept it and let it go* (26)… *I can more easily find a solution to my problems* (27)… *I can more easily observe my emotions with different eyes* (28)… *I face my negative emotions constructively* (29)… *if I feel a negative emotion, I recognize it, accept it and let it go* (30)… *I am more attentive to what happens around me moment by moment* (31)… *I am more aware of what happens around me moment by moment* (32)… *I can suspend my reactions and not act immediately* (33)… *I can relate with others in a more conscious and attentive manner* (34)… *when I interact with others I am less judgmental* (35)… *when I interact with others I can more easily talk about the emotions that emerge* (36)… *I can focus more on listening to what others say* (37)… *relationships with others are more profitable and productive*
**Benefits at work**
Thanks to the activities of visualization/concentration on the present… (38)… *during my work activities, I feel my body less stiff and tense* (39)… *during my work activities, I am more aware of my posture* (40)… *during my work activities, I can focus more on listening to what others say* (41)… *at work, relationships with others are more profitable and productive* (42)… *in difficult situations at work, I can suspend my reactions and not act immediately*

## References

[B1] Ackerman C. E. (2020). *Eleven Mindfulness Questionnaires, Scales and Assessments for Measuring Awareness.* Maastricht: Positive Psychology.

[B2] Baer R. A. Smith G. T. Allen K. B. (2004). Assessment of mindfulness by self-report: the Kentucky inventory of mindfulness skills. *Assessment* 11 191–206. 10.1177/1073191104268029 15358875

[B3] Baer R. A. Smith G. T. Hopkins J. Krietemeyer J. Toney L. (2006). Using self- report assessment methods to explore facets of mindfulness. *Assessment* 13 27–45. 10.1177/1073191105283504 16443717

[B4] Bentler P. M. (1990). Comparative fit indexes in structural models. *Psychol. Bull.* 107 238–246. 10.1037/0033-2909.107.2.238 2320703

[B5] Bergomi C. Tschacher W. Kupper Z. (2013b). The Assessment of Mindfulness with Self-Report Measures: Existing Scales and Open Issues. *Mindfulness* 4 191–202.

[B6] Bergomi C. Tschacher W. Kupper Z. (2013a). Measuring mindfulness: First steps towards the development of a comprehensive mindfulness scale. *Mindfulness* 4 18–32.

[B7] Birtwell K. Williams K. Van Marwijk H. Armitage C. Sheffield D. (2019). An Exploration of Formal and Informal Mindfulness Practice and Associations with Wellbeing. *Mindfulness* 10 89–99. 10.1007/s12671-018-0951-y 30662573 PMC6320743

[B8] Borgogni L. Galati D. Petitta L., and Centro Formazione Schweitzer. (2005). *The organizational checkup questionnaire. Adapted Italian manual.* Firenze: O.S. Organizzazioni Speciali.

[B9] Brennan P. A. Oeppen R. S. (2020). Editorial: safe healthcare teams during the coronavirus outbreak. *Br. J. Oral Maxillofacial Surg.* 58 254–255. 10.1016/j.bjoms.2020.03.011 32224009 PMC7195597

[B10] Brown K. W. Ryan R. M. (2003). The benefits of being present: mindfulness and its role in psychological well-being. *J. Pers. Soc. Psychol.* 84 822–848. 12703651 10.1037/0022-3514.84.4.822

[B11] Brown K. W. Ryan R. M. (2004). “Fostering healthy self-regulation from within and without: A Self-Determination Theory perspective,” in *Positive psychology in practice*, eds Linley P. A. Joseph S. (New York, NY: Wiley), 105–124.

[B12] Browne M. W. Cudeck R. (1993). “Alternative ways of assessing model fit,” in *Testing structural equation models*, eds Bollen K. A. Long J. S. (Newbury Park, CA: Sage), 136–162.

[B13] Buchheld N. Grossman P. Walach H. (2001). Measuring mind fulness in insight meditation (Vipassana) and meditation-based psychotherapy: The development of the Freiburg Mindfulness Inventory (FMI). *J. Meditat. Meditat. Res.* 2001 11–34.

[B14] Byrne B. M. (2006). *Structural Equation Modeling with EQS: Basic Concepts, Applications, and Programming*, 2nd Edn. Mahwah, NJ: Lawrence Erlbaum Associates.

[B15] Cardaciotto L. Herbert J. D. Forman E. M. Moitra E. Farrow V. (2008). The assessment of present-moment awareness and acceptance: The Philadelphia Mindfulness Scale. *Assessment* 15 204–223. 10.1177/1073191107311467 18187399

[B16] Chadwick P. Hember M. Symes J. Peters E. Kuipers E. Dagnan D. (2008). Responding mindfully to unpleasant thoughts and images: reliability and validity of the Southampton Mindfulness Questionnaire (SMQ). *Br. J. Clin. Psychol.* 47 451–455. 10.1348/014466508X314891 18573227

[B17] Chaskalson M. (2011). *The mindful workplace, developing resilient individuals and resonant organizations with MBRS.* London: John Wiley & Sons Itd.

[B18] Chou C. P. Bentler P. M. Satorra A. (1991). Scaled test statistics and robust standard errors for non-normal data in covariance structure analysis: A Monte Carlo study. *Br. J. Math. Statist. Psychol.* 44 347–357. 10.1111/j.2044-8317.1991.tb00966.x 1772802

[B19] Dorjee D. (2010). Kinds and dimensions of mindfulness: why it is important to distinguish them. *Mindfulness* 1 152–160. 10.1007/s12671-010-0016-3

[B20] Feldman G. Hayes A. Kumar S. Greeson J. Laurenceau J. P. (2007). Mindfulness and emotion regulation: The development and initial validation of the Cognitive and Affective Mindfulness Scale-Revised (CAMS-R). *J. Psychopathol. Behav. Assess.* 29:177. 10.1007/s12671-021-01784-5 34777622 PMC8576082

[B21] Geiser C. Götz T. Preckel F. Freund P. A. (2017). States and Traits. Theories, Models, and Assessment. *Eur. J. Psychol. Assess.* 33 219–223.

[B22] Giovannini C. Giromini L. Bonalume L. Tagini A. Lang M. Amadei G. (2014). The Italian Five Facet Mindfulness Questionnaire: A Contribution to its Validity and Reliability. *J. Psychopathol. Behav. Assess.* 36 415–423.

[B23] Glomb T. Duffy M. Bono J. Yang T. (2011). Mindfulness at Work. *Res. Pers. Hum. Resour. Manage.* 30 115–157.

[B24] Gunaratana H. (1990). *Mindfulness in plain English.* Boston: Wisdom Publications.

[B25] Hafenbrack A. (2017). Mindfulness Meditation as an On-The-Spot Workplace Intervention. *J. Bus. Res.* 75:17. 10.1016/j.jbusres.2017.01.017

[B26] Hanson J. Richardson Z. (2014). Mindfulness at work: A critical review. *Crit. Perspect. Bus. Manage.* 3 19–40.

[B27] Hatcher L. (1994). *A Step-by-Step Approach to Using the SAS^®^ System for Factor Analysis and Structural Equation Modeling.* Cary, N.C: SAS Institutte, Inc.

[B28] Hayes A. M. Feldman G. (2004). Clarifying the construct of mindfulness in the context of emotion regulation and the process of change in therapy. *Clin. Psychol. Sci. Pract.* 11, 255–262. 10.1093/clipsy.bph080

[B29] Hollon S. D. Kendall P. C. (1980). Cognitive self-statements in depression: Development of an automatic thoughts questionnaire. *Cognit. Therapy Res.* 4 383–395.

[B30] Hu L. Bentler P. M. (1999). Cutoff criteria for fit indexes in covariance structure analysis: Conventional criteria versus new alternatives. *Struct. Equat. Model.* 6 1–55.

[B31] Kabat-Zinn J. (1990). *Full catastrophe living: How to cope with stress, pain and illness using mindfulness meditation.* New York, NY: Bantam Dell.

[B32] Kabat-Zinn J. (2003). Mindfulness-based interventions in context: Past, present, and future. *Clin. Psychol. Sci. Pract.* 10 144–156.

[B33] Kabat-Zinn J. (2005). *Coming to our senses: healing ourselves and the world through mindfulness.* New York, NY: Hyperion.

[B34] Kabat-Zinn J. (2020). The Suspension of Distraction. *Mindfulness* 11 2237–2238.

[B35] Langer E. J. Moldoveanu M. (2000). Mindfulness research and the future. *J. Soc. Iss.* 56 1–9.

[B36] Lau M. A. Bishop S. R. Segal Z. V. Buis T. Anderson N. D. Carlson L. (2006). The Toronto Mindfulness Scale: Development and validation. *J. Clin. Psychol.* 62 1445–1467. 10.1002/jclp.20326 17019673

[B37] Little T. D. (2013). *Longitudinal Structural Equation Modeling.* Guilford Press.

[B38] Little T. D. Rhemtulla M. Gibson K. Schoemann A. M. (2013). Why the items versus parcels controversy needn’t be one. *Psychol. Methods* 18 285–300. 10.1037/a0033266 23834418 PMC3909043

[B39] Luken M. Sammons A. (2016). Systematic review of mindfulness practice for reducing job burnout. *Am. J. Occupat. Therapy* 70 7002250020p1–7002250020p10 10.5014/ajot.2016.016956 26943107 PMC4776732

[B40] Maslach C. (2003). Job burnout: new directions in research and intervention. *Curr. Dir. Psychol. Sci.* 12, 189–192. 10.1111/1467-8721.01258

[B41] Maslach C. Leiter M. P. (2008). Early predictors of job burnout and engagement. *J. Appl. Psychol.* 93, 498–512. 10.1037/0021-9010.93.3.498 18457483

[B42] Maslach C. Leiter M. P. (2016). “Burnout,” in *Handbook of stress: Vol. 1. Stress: Concepts, cognition, emotion, and behavior*, ed. Fink G. (Amsterdam: Elsevier Academic Press), 351–357.

[B43] McVay J. C. Kane M. J. (2009). Conducting the train of thought: Working memory capacity, goal neglect, and mind wandering in an executive-control task. *J. Exp. Psychol.* 35 196–204. 10.1037/a0014104 19210090 PMC2750806

[B44] Meade A. W. Johnson E. C. Braddy P. W. (2008). Power and sensitivity of alternative fit indices in tests of measurement. *J. Appl. Psychol.* 93 568–592. 10.1037/0021-9010.93.3.568 18457487

[B45] Medvedev O. N. Krägeloh C. U. Narayanan A. Siegert R. J. (2017). Measuring mindfulness: Applying generalizability theory to distinguish between state and trait. *Mindfulness* 8 1–11.

[B46] Muthèn B. Kaplan D. (1985). A comparison of some methodologies for the factor analysis of non-normal Likert variables. *Br. J. Math. Statist. Psychol.* 38 171–189.

[B47] Muthén L. K. Muthén B. O. (1998-2017). *Mplus User’s Guide*, 8th Edn. Los Angeles, CA: Muthén & Muthén.

[B48] Petitta L. Di Cave F. (2011). “Emotional contagion at work and group performance,” in *Advances in Understanding the Links of Emotions and Context. Symposium Presented at the 26th Conference SIOP*, eds Petitta L. Diefendorff J. (Chicago: Society for Industrial and Organizational Psychology), 14–16.

[B49] Pirson M. Langer E. J. Bodner T. Zilcha-Mano S. (2012). “The development and validation of the Langer Mindfulness Scale: Enabling a socio-cognitive perspective of mindfulness in organizational contexts,” in *Fordham University Schools of Business Research Paper* (New York, NY: Fordham University).

[B50] Schaufeli W. B. Leiter M. P. Maslach C. Jackson S. E. (1996). “MBI-General Survey,” in *Maslach Burnout Inventory*, 3rd Edn, eds Maslach C. Jackson S. E. Leiter M. P. (Palo Alto, CA: Consulting Psychologist Press).

[B51] Shapiro S. L. Carlson L. E. Astin J. A. Freedman B. (2006). Mechanisms of mindfulness. *J. Clin. Psychol.* 62 373–386. 10.3390/brainsci12020285 16385481

[B52] Siegel D. J. (2007). Mindfulness training and neural integration: differentiation of distinct streams of awareness and the cultivation of well-being. *Soc. Cognit. Affect. Neurosci.* 2 259–263. 10.1093/scan/nsm034

[B53] Siegel D. J. (2010). *The mindful therapist: A clinician’s guide to mindsight and neural integration.* New York, NY: WW Norton & Company.

[B54] Siegel D. J. (2018). *Aware: The science and practice of presence.* New York, NY: Tarcher/Perigee Penguin Random House.

[B55] Singh N. Lancioni G. Hwang Y. (2021). “Mindfulness Care Giving and Support for Anger and Aggression Management,” in *Applied Behavior Analysis Treatment of Violence and Aggression in Persons with Neurodevelopmental Disabilities*, ed. Luiselli J. (New York, NY: Springer), 189–202. 10.1002/14651858.CD003406.pub4

[B56] Solloway S. G. Fisher W. P. Jr. (2007). Mindfulness practice: A Rasch variable construct innovation. *J. Appl. Measur.* 8 359–372. 18250523

[B57] Spector P. E. (2006). Method variance in organizational research: truth or urban legend? *Organ. Res. Methods* 9, 221–232. 10.1177/1094428105284955

[B58] Spector P. E. Brannick M. T. (2011). Methodological Urban Legends: The Misuse of Statistical Control Variables. *Organiz. Res. Methods* 14 287–305.

[B59] Tan C. (2014). “Search inside yourself: the unexpected path to achieving success, happiness (and world peace),” in *First HarperCollins paperback edition* (New York, NY: HarperOne).

[B60] Tanay G. Bernstein A. (2013). State Mindfulness Scale (SMS): Development and initial validation. *Psychol. Assess.* 25 1286–1299. 10.1037/a0034044 24059475

[B61] Walach H. Buchheld N. Buttenmüller V. Kleinknecht N. Schmidt S. (2006). Measuring mindfulness: The Freiburg Mindfulness Inventory (FMI). *Pers. Individ. Differ.* 40 1543–1555.

[B62] Wallace B. A. (1999). The Buddhist tradition of Samatha: Methods for refining and examining consciousness. *J. Conscious. Stud.* 6 175–187.

[B63] Walsh R. Shapiro S. L. (2006). The meeting of meditative disciplines and western psychology: A mutually enriching dialogue. *Am. Psychol.* 61 227–239. 10.1037/0003-066X.61.3.227 16594839

[B64] Westland C. J. (2010). Lower bounds on sample size in structural equation modeling. *Electr. Commerce Res. Applicat.* 9 476–487. 10.1007/s11121-014-0489-8 24752569 PMC4207737

[B65] Xiao Q. Yue C. He W. Yu J. Y. (2017). The Mindful Self: A Mindfulness-Enlightened Self-view. *Front. Psychol.* 8:1752. 10.3389/fpsyg.2017.01752 29081754 PMC5645519

